# Association between maternal PM_2.5_ exposure and congenital heart defects in offspring: a time-series study

**DOI:** 10.3389/fpubh.2026.1782443

**Published:** 2026-05-04

**Authors:** Qunying Li, Yunyun Wu, Zihao Wang, Yi Li, Deqiang Mao, Qi Zhang, Ke Chen

**Affiliations:** 1 Chongqing Municipal Center for Disease Control and Prevention, Chongqing Institute of Preventive Medicine, Chongqing, China; 2 Department of Medical Services, The First Affiliated Hospital of Chongqing Medical University, Chongqing, China

**Keywords:** ambient air pollution, congenital heart defects, distributed lag nonlinear model, fine particulate matter, gestational exposure, time series analysis

## Abstract

**Background:**

Maternal exposure to air pollutants are associated with congenital heart defects (CHDs), yet fundamental questions remain unclear: which pollutant matters most, which exposure window is most critical, and whether susceptibility differs by offspring sex.

**Objective:**

To examine the association between maternal PM_2.5_ exposure during periconception (from 4 weeks before conception to 28 weeks of gestation) and offspring CHD risk in urban districts of Chongqing (2018 ~ 2021), and to identify the key exposure windows.

**Methods:**

Data on birth defects and air quality in urban Chongqing were utilized. The association between PM_2.5_ exposure and congenital heart disease was assessed using the Distributed Lag Nonlinear Model (DLNM) time-series analysis.

**Results:**

The average concentration of PM_2.5_ in urban Chongqing from 2018 to 2021 was 37.27 μg/m^3^. A total of 884 cases of congenital heart disease were monitored. We observed significant increasing effects between PM_2.5_ exposure during gestational weeks 6 to 27 and risk of CHDs (RR = 1.05, 95% CI: 1.01 ~ 1.09) per a 10 μg/m^3^ change in PM_2.5_ concentration. Sex-stratified analysis revealed that male offspring exhibited greater susceptibility from preconception week 3 to gestational week 24, with the strongest effect at gestational week 11 ~ 12 (RR = 1.10, 95% CI: 1.04 ~ 1.16). For congenital malformations of cardiac septa (Q21), the critical susceptibility windows were gestational weeks 0 ~ 26, with the strongest effect at gestational week 13 (RR = 1.06, 95% CI: 1.02 ~ 1.11). While, congenital malformations of great arteries (Q25) demonstrated susceptibility from preconception week 3 to gestational week 21, with the strongest effect at gestational week 10 (RR = 1.11, 95% CI: 1.04 ~ 1.19).

**Significance:**

Maternal exposure to PM_2.5_, especially during the preconception and the first trimester, increases the risk of certain types of CHDs in offspring, and male offspring exhibited greater susceptibility.

**Impact:**

Based on data from Chongqing (2018 ~ 2021), this study used DLNM method to identify that maternal exposure to PM_2.5_ between gestational weeks 6 ~ 27 significantly increased the risk of CHDs, including cardiac septal defects (Q21) and great artery malformations (Q25). Male offspring showed higher susceptibility, with the strongest effect in gestational weeks 11 ~ 12. Associations were modified after adjusting for co-pollutants (SO₂, NO₂, CO, O₃) in two-pollutant models.

## Introduction

Air pollution, especially atmospheric particulate matter pollution, is a major global public health problem. The Global Burden of Disease Study 2021 pointed out that air particulate matter pollution seriously threatens human health and is the top health hazard factor. Approximately 8.0% of disability-adjusted life years (DALYs) worldwide are related to air particulate matter pollution (1). Fine particulate matter (PM_2.5_), defined as particles with a diameter of ≤2.5 μm, is a key atmospheric pollutant. Due to its small size, PM2.5 can bypass the nasal and upper respiratory tract defenses, penetrate deep into the lower respiratory tract, and reach the alveoli, thereby causing systemic harm. In response to these risks, the Global Air Quality Guidelines updated by the World Health Organization in 2021 tighten the annual average concentration limit of PM_2.5_ to 5 μg/m^3^. Despite this standard, over 94% of the global population resides in areas where PM_2.5_ levels exceed this threshold, leaving the majority still vulnerable to its health impacts (2–4).

Congenital heart disease (CHD) has a global incidence of approximately 9.41 per 1,000 live births (5), imposing a substantial disease burden that disproportionately affects children, especially infants under one year of age (6). China has the world’s largest number of CHD cases (7), with a rapid increase reported between 2005 and 2019 (8).

Emerging epidemiological evidence suggests a link between maternal exposure to air pollution and congenital heart defects (9, 10). Air pollutants may adversely affect pregnancy outcomes through mechanisms such as endocrine disruption, oxidative stress, inflammation, and DNA damage (11–13). Animal studies further support that in utero exposure to air pollutants can induce fetal malformations (14, 15). While previous research has largely focused on preterm birth and low birth weight, evidence regarding birth defects remains limited and inconsistent. Several epidemiological studies and meta-analyses have reported that in utero exposure to ambient air pollution potentially increase the risk of congenital heart defects in newborns (16, 17). Most studies assign exposure based on average pollutant levels during weeks 3–8 or the first trimester of pregnancy, both of which are critical windows for cardiac development. However, such approaches may ignore temporal changes in exposure, as well as interactions between different pollutants, potentially masking true associations. Furthermore, the majority of evidence comes from high-income countries, with scarce data from low- and middle-income regions like China.

China’s rapid economic growth in recent decades has been accompanied by severe air pollution. Chongqing, characterized by heavy industry and basin topography, is among the country’s most polluted cities. Industrial production is a major source of urban PM_2.5_ in this region, as reflected in local environmental reports from 2018 to 2021. However, few studies have examined the relationship between prenatal PM_2.5_ exposure and CHD in Chongqing.

This study aims to address this gap by using a distributed lag non-linear model to analyze the association between maternal PM_2.5_ exposure during pregnancy and congenital heart disease in offspring. We also seek to identify susceptible exposure windows, with the goal of providing evidence-based health guidance for pregnant women, optimizing prenatal care, and mitigating the adverse effects of environmental factors on fetal development.

## Materials and methods

### Congenital heart disease data

This study utilized data from the Chongqing Birth Defect Surveillance System, covering the period from January 1, 2018, to December 31, 2021. The database encompassed nine urban districts namely Yuzhong District, Jiangbei District, Shapingba District, Jiulongpo District, Nan’an District, Dadukou District, Beibei District, Yubei District and Banan District in Chongqing. The Birth Defect Surveillance System is a national, hospital-based, computer-based standardized database that accurately and prospectively collects birth defects. The diagnosis of Birth Defects was performed by qualified doctors of medical institutions based on the “International Statistical Classification of Diseases and Related Health Problems, Tenth Edition” (ICD-10). The database contained maternal and infant characteristics such as infant sex, maternal age, ethnicity, educational level, gravidity, and parity. Cases with CHDs were included based on ICD-10 codes Q20–Q26, which encompass congenital malformations of cardiac chambers, septa, valves, great arteries, and great veins (18). We excluded cases involving multiple pregnancies, maternal age outside the 16–50 range, prenatal exposure to known teratogens, medication use during early pregnancy, or presence of genetic abnormalities.

This study was approved by the Ethics Committee of Chongqing Center for Disease Control and Prevention (KY-2023-044-1).

### Environmental data

Data on air pollution, including levels of PM_2.5_, PM < 10 μm in aerodynamic diameter (PM_10_), sulfur dioxide (SO_2_), nitrogen dioxide (NO_2_), carbon monoxide (CO), and ozone (O_3_), were obtained from the Chongqing Ecology and Environment Bureau for the period from January 1, 2017, to December 31, 2021. The data were collected from 22 state and municipal monitoring stations across the nine urban districts. Daily exposure levels for all pollutants except O₃ were represented by the 24-h average concentration; for O₃, we used the 8-h maximum average. The concentration units were μg/m^3^ for all pollutants except CO, which was measured in mg/m^3^. PM10 and PM_2.5_ were monitored using a continuous *β*-ray method, SO₂ and O₃ by ultraviolet fluorescence, and NO₂ by chemiluminescence.

The meteorological monitoring data are from the Chongqing Meteorological Bureau, including daily average temperature, daily average relative humidity, daily average pressure and daily average wind speed. The missing rate of all air pollution and meteorological indicators was less than 0.01%, and the missing value was filled by the arithmetic average of the two days before and after.

### Statistical analysis

Descriptive analysis was conducted on quantitative data using indicators such as mean ± standard deviation (sd), percentiles (P25, P50, P75), maximum value and minimum value. Statistical analysis Spearman’s correlation coefficient was applied to explore the correlation between air pollutants and meteorological factors.

Congenital heart defects in babies born every week are a low probability event, which can be considered to conform to the Poisson distribution. Therefore, we used the discrete Poisson-like distribution connection function DLNM to analyze the relationship between the number of congenital heart defects per week and the concentration of ambient air pollutants in pregnancy. Based on the birth defects surveillance data, the date of conception was calculated by the date of birth and gestational age. The environmental exposure data were collected by the date of conception and included in the model by the week. In order to control the influence of meteorological factors on pregnant women, meteorological exposure data were also included in the model by using weekly average data, and the influence of long-term trend and period effect inherent in time series analysis on the results was controlled. The specific formula of the model is as follows:


Yt~Poisson(μt)

$$\begin{array}{l} \log(\mathrm{\mu t})=\alpha +\beta T_{t,1}+\mathrm{ns}(\text{time},\mathrm{df})+\mathrm{ns}(\text{temp},\mathrm{df})+\mathrm{ns}(\mathrm{fs},\mathrm{df})+\\ \mathrm{ns}(\mathrm{sd},\mathrm{df})+\mathrm{ns}(\mathrm{qy},\mathrm{df})=\alpha +\beta T_{t,1}+\text{COVs} \end{array}$$

In the formula, μt is the number of CHDs in week t; t is the number of weeks observed; *α* is the intercept; and *β* is the regression coefficient; l represents the number of lagging weeks; Tt,1 refers to the concentration of pollutants at different lag times; df represents the degree of freedom; ns refers to natural smooth function; temp refers to average temperature; fs refers to the average wind speed; sd refers to the average humidity; qy refers to the average air pressure; COVs refers to the confounding factors to be adjusted.

We constructed a large time series by arranging the pregnancy dates of the birth defects in order of their occurrence. The lag function was used to investigate pollutant exposure during pregnancy trimesters.

The relative risk (RR) and 95% confidence interval (95% CI) were employed as effect measures to quantify the probability of an increase in the number of CHDs associated with each 10 μg/m^3^ increment in the weekly average concentration of PM_2.5_.

The R software version 3.6.0 was used for data cleaning and statistical analysis, and the “DLNM” package was used to fit the model. The significance level for hypothesis testing was set at *α* = 0.05.

## Results

### Basic characteristics of patients

A total of 884 newborns were diagnosed with congenital heart defects, comprising 428 males and 456 females. Case numbers distributed by season were 241 in spring, 244 in summer, 200 in autumn, and 199 in winter. The most prevalent subtypes were cardiac septal defects (Q21, 85.75%) and congenital malformations of the great arteries (Q25, 42.99%).

### Air pollutants and meteorological factors

**Figure 1:**
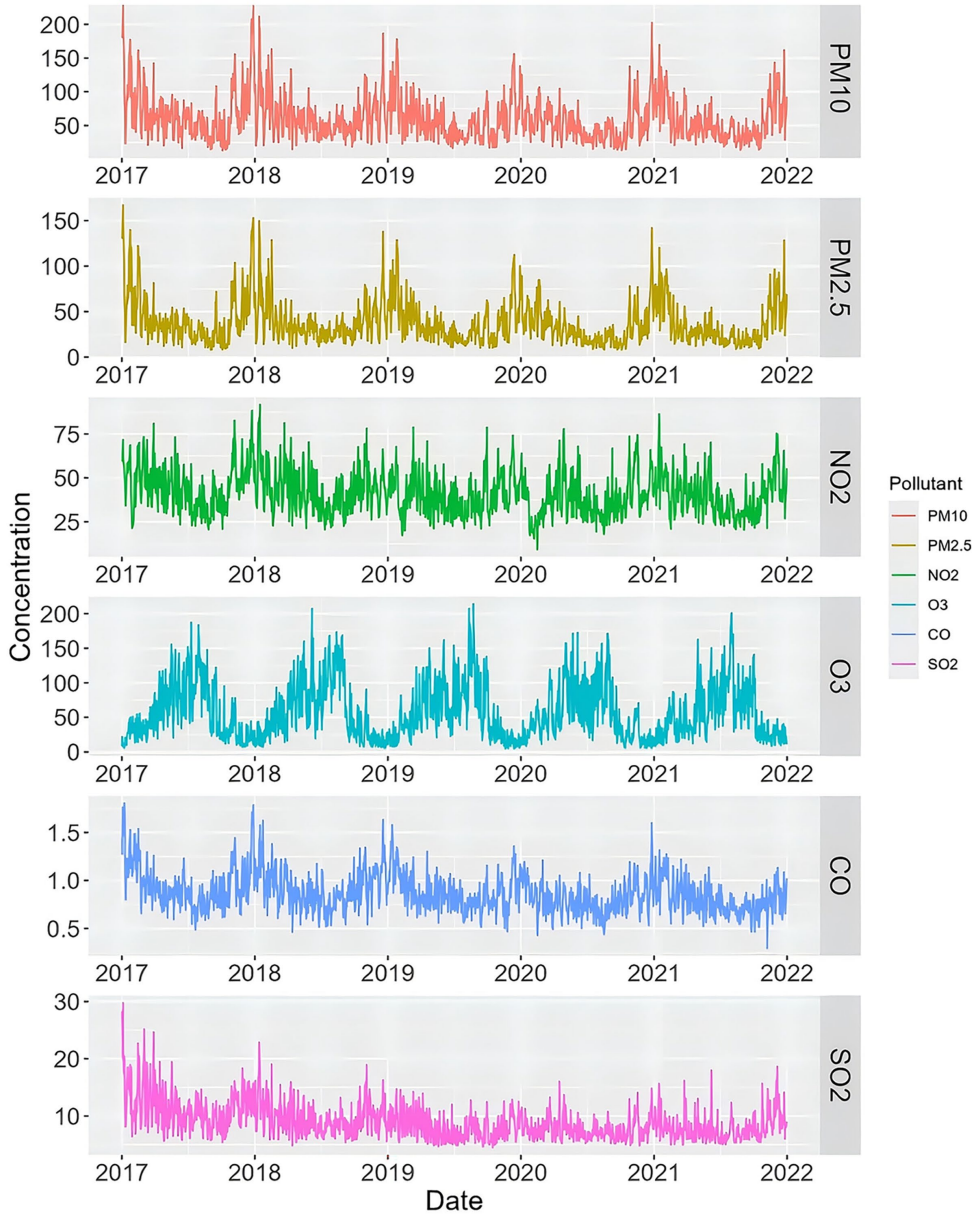
The time series of air pollutant concentration in nine urban districts among Chongqing from 2017 to 2021.

Basic characteristics of meteorological factors, and air pollution are displayed in Table 1. The average concentration of PM_2.5_ in the nine districts of Chongqing from 2018 to 2021 was 37.27 μg/m^3^ (range, 7.83 to 166.98 μg/m^3^, Table 2). There were 144 days with PM_2.5_ exceeding the secondary concentration limit (75 μg/m^3^) of National Environmental Air Quality Standard (GB3095-2012), accounting for 7.88%. The concentration of air pollutants fluctuated with seasons, with PM_2.5_ being higher in winter and lower in summer. However, in the long run, PM_2.5_ concentration shows a downward trend year by year. Figure 1 illustrates the trend of atmospheric pollutant concentrations.

**Table 1:** Characteristics of CHDs in the nine urban districts of Chongqing from 2018 to 2021.

Characteristic	*N*	Pre (%)
Child’s sex		
Male	428	48.42
Female	456	51.58
Season of birth		
Spring	241	27.26
Summer	244	27.60
Autumn	200	22.62
Winter	199	22.51
Maternal age (year)		
<35	771	87.22
≥35	113	12.78
Gravidity		
≤2	616	69.68
>2	268	30.32
Gestational age, weeks		
<37w	221	25.00
≥37w	663	75.00
Birth weight (g)		
<2,500	179	20.25
≥2,500	705	79.75
Types of CHDs		
Q20	5	0.57
Q21	758	85.75
Q22	23	2.60
Q23	2	0.23
Q24	26	2.94
Q25	380	42.99
Q26	12	1.36

Q20–Q26 represent congenital heart disease categories coded according to the International Classification of Diseases, 10th Revision (ICD-10), with specific classifications as follows: Q20: Congenital malformations of cardiac chambers and connections; Q21: Congenital malformations of cardiac septa; Q22: Congenital malformations of pulmonary and tricuspid valves; Q23: Congenital malformations of aortic and mitral valves; Q24: Other congenital malformations of heart; Q25: Congenital malformations of great arteries; Q26: Congenital malformations of great veins.

**Table 2:** Distribution of air pollutants and meteorological factors in nine urban districts among Chongqing from 2018 to 2021.

Variables	$\bar{x}\pm s$	Min	*P*_25_	Median	*P*_75_	Max
Air pollutants						
PM_2.5_/μg/m^3^	37.27 ± 23.74	7.83	20.81	30.85	45.73	166.98
PM_10_/μg/m^3^	59.85 ± 32.55	12.86	36.46	53.07	74.19	228.17
SO_2_/μg/m^3^	8.94 ± 3.08	4.48	6.71	8.22	10.49	29.37
NO_2_/ μg/m^3^	41.44 ± 12.56	9.29	31.77	39.79	49.50	91.70
CO/mg·m^−3^	0.87 ± 0.21	0.32	0.73	0.84	0.99	1.80
O_3_/mg·m^−3^	57.53 ± 41.14	4.57	24.78	45.82	83.74	212.83
Meteorological factors						
Mean pressure (hpa)	983.18 ± 8.69	964.3	975	983.8	990.05	1,006
Mean temperature (°C)	19.35 ± 7.77	4.2	12.2	18.9	25.6	36.5
Relative humidity (%)	75.29 ± 11.34	38	68	76.8	84	97
Wind speed (m·s^−1^)	1.52 ± 0.69	0.1	1.1	1.4	1.7	9.9

Spearman’s correlation coefficients among air pollutants and meteorological factors are listed in Table 3. PM_2.5_ was positively correlated with SO₂, NO₂, CO, and mean pressure, and negatively correlated with O₃, mean temperature, and relative humidity. NO₂ showed positive associations with CO and mean pressure, but negative associations with O₃, mean temperature, and relative humidity. CO was positively correlated with mean pressure and relative humidity, and negatively correlated with O₃ and mean temperature. O₃ was positively associated with mean temperature, and negatively associated with mean pressure and relative humidity.

**Table 3:** Spearman’s correlation coefficients between air pollutants and meteorological factors in nine urban districts among Chongqing from 2018 to 2021.

Variables	SO_2_	NO_2_	CO	O_3_	Mean pressure	Mean temperature	Relative humidity
PM_2.5_	0.62*	0.70*	0.74*	−0.25*	0.41*	−0.48*	−0.10*
SO_2_	1.00	0.63*	0.50*	0.05	0.10*	−0.07*	−0.47*
NO_2_		1.00	0.71*	−0.18*	0.30*	−0.27*	−0.10*
CO			1.00	−0.34*	0.30*	−0.38*	0.14*
O_3_				1.00	−0.68*	0.76*	−0.66*
Mean pressure					1.00	−0.88*	0.35*
Mean temperature						1.00	−0.40*
Relative humidity							1.00

^*^p < 0.05.

### PM_2.5_ exposure and congenital heart disease

**Figure 2:**
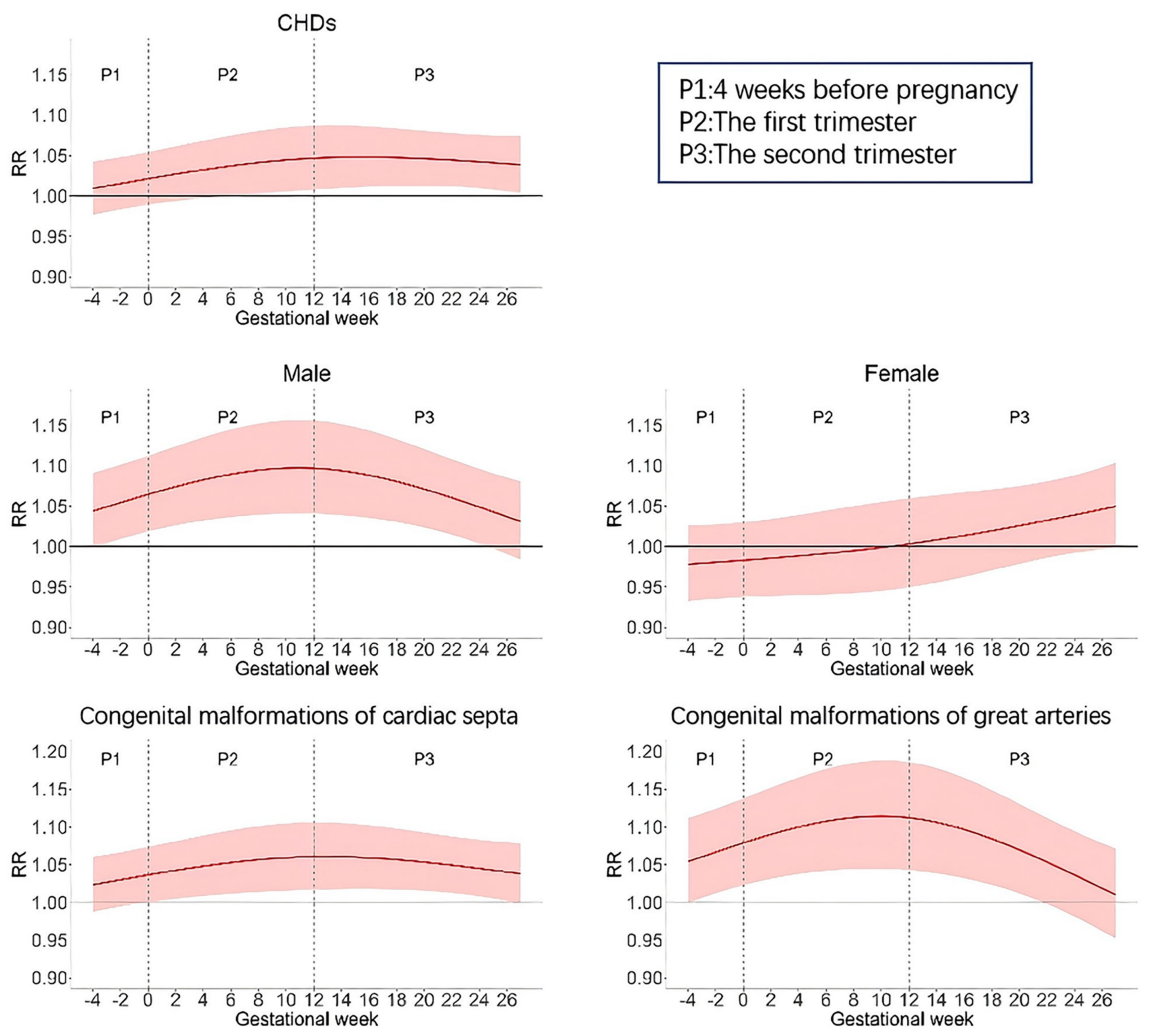
The relative risk of CHDs occurrence at different gestational weeks for a 10 μg/m^3^ increase of PM2.5 in single pollutant model.

We incorporated average temperature and relative humidity into the distributed lag nonlinear model to adjust for the influence of meteorological conditions, while controlling for the time trend. Then, a single pollution model was fitted to analyze the impact of prenatal exposure to atmospheric PM_2.5_ alone on the outcome of congenital heart disease in the offspring (Figure 2). Overall, exposure to atmospheric PM_2.5_ during the 6th to 27th weeks of pregnancy increases the risk of congenital heart disease in the offspring (*p* < 0.05). The risk is highest at the 15th week. For every 10 μg/m^3^ increase in PM_2.5_ concentration, the corresponding RR (95% CI) is 1.05 (1.01–1.09). For male offspring, the PM_2.5_ effect window period is from 3 weeks before pregnancy to the 24th week of pregnancy. The effect is strongest during the 11th-12th weeks of pregnancy, with an RR (95% CI) of 1.10 (1.04–1.16). However, the effect is not significant for female offspring. For congenital cardiac septal malformations (Q21), the effect window period is from the 0th to the 26th week of pregnancy, with the strongest effect occurring at the 13th week, and the RR (95% CI) is 1.06 (1.02–1.11); for congenital arterial anomalies (Q25), the effect exists from the 3rd week before pregnancy to the 21st week of pregnancy, and the strongest effect occurs at the 10th week of pregnancy, with the RR (95% CI) being 1.11 (1.04–1.19).

We defined the following exposure windows: the 4 weeks before pregnancy, the first trimester (gestational weeks 0–12), and the second trimester (gestational weeks 13–28). Table 4 presents the cumulative relative risks (RRs) of congenital heart disease in offspring associated with PM_2.5_ exposure during these periods.

**Table 4:** The relative risk of CHDs occurrence at different gestational weeks for a 10 μg/m^3^ increase of PM2.5 in single pollutant model.

Group	Window period	RR (4 weeks before pregnancy)	RR (The first trimester)	RR (The second trimester)	RR (From 4 weeks before pregnancy to the second trimester)
Total	Gestational weeks 6 to 27	1.06 (0.93 ~ 1.20)	1.51 (0.99 ~ 2.30)	2.02 (1.18 ~ 3.48)	3.23 (1.08 ~ 9.61)
Offspring gender					
Male	Gestational weeks 3 to 24	1.22 (1.03 ~ 1.46)	2.66 (1.48 ~ 4.78)	2.94 (1.38 ~ 6.25)	9.55 (2.10 ~ 43.50)
Female	—	0.92 (0.76 ~ 1.11)	0.897 (0.49 ~ 1.66)	1.48 (0.67 ~ 3.26)	1.22 (0.25 ~ 6.03)
Types of CHDs					
Q21	Gestational weeks 0 to 26	1.13(0.98 ~ 1.30)	1.84(1.14 ~ 2.97)	2.15(1.18 ~ 3.92)	4.48(1.32 ~ 15.17)
Q25	From 3 weeks before pregnancy to Gestational weeks 21	1.31(1.06 ~ 1.62)	3.31(1.60 ~ 6.84)	2.64(1.07 ~ 6.53)	11.45(1.82 ~ 72.06)

The overall cumulative RR for CHDs from 4 weeks before pregnancy through the second trimester was 3.23 (95% CI: 1.08–9.61). Among the three windows, only the second trimester showed a statistically significant cumulative RR of 2.02 (95% CI: 1.18–3.28). In sex-stratified analyses, the total cumulative RR for male offspring was 9.55 (95% CI: 2.10–43.50), with the strongest effect observed in the second trimester, followed by the first trimester and the pre-pregnancy period. For female offspring, no statistically significant associations were found. For congenital cardiac septal malformations (Q21), the total cumulative RR of PM_2.5_ exposure from 4 weeks before pregnancy to the second trimester was 4.48 (95% CI: 1.32–15.17). Among them, the cumulative RR in the first trimester and the second trimester was statistically significant, and the cumulative RR in the second trimester was 2.15 (95% CI: 1.18–3.92), which was the strongest. For congenital arterial anomalies (Q25), the total cumulative RR from 4 weeks before pregnancy to the second trimester was 11.45 (95% CI: 1.82–72.06). All three exposure windows showed significant associations, with the strongest effect in the first trimester.

We further developed two-pollutant models by individually adding SO₂, O₃, NO₂, and CO to the base single-pollutant model. Results showed that after adjusting for NO₂, the association between prenatal PM_2.5_ exposure and CHDs was strengthened, with an earlier susceptible window. In contrast, adjustment for O₃ attenuated the effect of PM_2.5_ and shortened the susceptible window, mainly concentrating in the second trimester. The association disappeared when adjusting for SO₂ or CO, as well as in the multi-pollutant model including all contaminants (Table 5).

**Table 5:** The relative risk of CHDs occurrence at different gestational weeks for a 10 μg/m^3^ increase of PM2.5 in multi-pollutant model.

Model	Adjusted pollutant	Window period	RR (Gestational week with the highest RR)	RR (4 weeks before pregnancy)	RR (The first trimester)	RR (The second trimester)	RR (From 4 weeks before pregnancy to the second trimester)
Single pollutant model	PM_2.5_	Gestational weeks 6 to 27	1.05 (1.01 ~ 1.09)	1.06 (0.93 ~ 1.20)	1.51 (0.99 ~ 2.30)	2.02 (1.18 ~ 3.48)	3.23 (1.08 ~ 9.61)
Double pollutants model 1	PM_2.5_ + NO_2_	From 2 weeks before pregnancy to 21 weeks after pregnancy	1.07 (1.01 ~ 1.14)	1.16 (0.99 ~ 1.36)	2.10 (1.14 ~ 3.87)	2.35 (1.01 ~ 5.47)	5.72 (1.14 ~ 28.68)
Double pollutants model 2	PM_2.5_ + O_3_	Gestational weeks 17 to 27	1.044 (1.01 ~ 1.08)	1.036 (0.89 ~ 1.20)	1.38 (0.84 ~ 2.29)	1.964 (1.03 ~ 3.76)	2.82 (0.77 ~ 10.34)
Double pollutants model 3	PM_2.5_ + CO	—	1.03 (0.99 ~ 1.07)	1.12 (0.97 ~ 1.30)	1.27 (0.74 ~ 2.19)	1.24 (0.59 ~ 2.62)	1.77 (0.42 ~ 7.44)
Double pollutants model 4	PM_2.5_ + SO_2_	—	1.03 (0.99 ~ 1.08)	1.04 (0.88 ~ 1.21)	1.37 (0.88 ~ 2.13)	1.52 (0.79 ~ 2.91)	2.15 (0.62 ~ 7.52)
All pollutants model	PM_2.5_ + SO_2_ + NO_2_ + CO + O_3_	—	1.05 (0.97 ~ 1.12)	1.11 (0.89 ~ 1.39)	1.62 (0.77 ~ 3.41)	1.37 (0.45 ~ 4.10)	2.45 (0.31 ~ 19.43)

Besides air pollutants, meteorological factors (including mean pressure, mean temperature, relative humidity and wind speed) and time trend are also adjusted in all models.

## Discussion

During the past few decades, CHDs are the most common severe congenital anomalies and the leading cause of infant mortality due to congenital anomalies, and the aetiologies are unknown for the majority of these defects (14, 19, 20). Epidemiological studies reported that prenatal exposure to air pollutants has a direct negative impact on birth outcomes (14, 21, 22). In this study conducted among Chinese women and infants exposed to a very high level of pollution, we observed an increased risk of CHDs, particularly Congenital malformations of great arteries, with increasing PM_2.5_ exposure (Table 5).

With the rapid development of urbanization and industrialization, most parts of China are suffering from serious air pollution in the past few decades. The average daily concentration of PM_2.5_ in the central urban area of Chongqing was 37.27 μg/m^3^ from 2018 to 2021, which is slightly higher than the national average. In the long-term trend, PM_2.5_ pollution is decreasing year by year, but it is still far beyond the 5 μg/m^3^ level recommended by WHO.

In this study, distributed lag non-linear model was used to analyze the atmospheric PM_2.5_ before and during pregnancy in the central urban area of Chongqing from 2018 to 2021. Association between exposure and congenital heart disease in offspring. More and more scholars have noticed that the susceptibility window of air pollution may not be completely consistent with the life cycle of heart development (14, 23). Therefore, we divided pregnancy into longer exposure periods, such as 4 weeks before pregnancy, the first trimester (Gestation from 0 to 12 weeks) and the second trimester (Gestation from 13 to 28 weeks). The results showed that the risk of congenital heart defects was 3.23 (95% CI: 1.08–9.61) for every 1 μg/m^3^ increase in PM_2.5_ in gestational weeks 6 to 27, which made an important supplement to the conclusion we arrived at previously when analyzing the single factor of PM_2.5_ (23).

Maternal PM_2.5_ exposure had a greater effect on congenital heart disease in male offspring, but not in female offspring, which was consistent with the fact that the birth detection rate of congenital heart disease in males was higher than that in females in China (4.2‰ vs. 3.5 ‰)[8]. This may be related to the expression of sex chromosomal-related genes and their interaction with hormonal effects during early development (24). There are also animal experimental studies showing that the offspring of maternal mice exposed to PM_2.5_ have increased pathological damage to the heart, and the damage is more pronounced in the male group than in the female group (25).

The effects of different types of birth defects on PM_2.5_ also vary, and this conclusion has also been verified in other studies (25, 26). Owing to variations in factors such as study design, exposure assessment methods, and pollutant characteristics, some studies—notably those that subdivided congenital heart defects into specific types—have even reported an inverse association between maternal PM2.5 exposure and CHDs. (27–29). The results of National Birth Defects Prevention Study (NBDPS) in the United States revealed that exposure to fine particulate matter was positively associated with hypoplastic left heart syndrome (HLHS) and negatively associated with atrial septal defects (30). The results of this study indicate that PM_2.5_ can increase the risk of congenital cardiac septal malformations (including ventricular septal defect, atrial septal defect, patent foramen ovale, tetralogy of Fallot, etc.) and congenital large artery malformations (including patent ductus arteriosus, aortic stenosis, etc.)The cumulative RR for congenital septal heart defects (Q21) was 4.48 (95% CI: 1.32–15.17), with a window period from Gestational weeks 0 to 26, and the effect was strongest at 13 weeks of gestation, with RR (95% CI) being 1.06 (1.02–1.11). For congenital great artery defects (Q25), the effect existed from 3 weeks before conception to Gestational weeks 21, with the strongest effect at Gestational weeks 10, with RR (95% CI) being 1.11 (1.04–1.19). This may be related to the different development times of various structures of the heart.

The research shows that after correcting for the coexisting pollutants SO_2_, NO_2_, CO, and O_3_ using the dual-pollution model, the effects of pre-pregnancy and during-pregnancy PM_2.5_ exposure on the occurrence of congenital heart disease in offspring changed. Specifically, the effect of PM_2.5_ became stronger when correcting for NO_2_, while the effect weakened or even disappeared when correcting for O_3_, SO_2_, and CO. It is similar to one study about CO and SO_2_ has a protective effect on CHDs (31). Most research results indicate that there is a positive correlation between NO_2_ and PM_2.5_ and congenital heart disease, but the relationship between SO_2_, CO, O_3_ and congenital heart disease is controversial (31–33). Studies have shown that components of PM_2.5_, such as sulfates, nitrates, and ammonium salts, have a significant impact on the occurrence of congenital heart disease in offspring (23). Therefore, the effects of prenatal exposure to PM_2.5_ and other atmospheric pollutants on the occurrence of congenital heart disease in offspring may have synergistic effects, suggesting that the influence of other pollutants should be considered when studying the health effects of PM_2.5_.

This study has the following limitations. Firstly, outdoor levels may not be as good of a surrogate for personal exposure levels, the “outdoor-indoor-internal” exposure matrix will provide stronger support for exposure assessment. Secondly, residual classification errors may lead to incorrect classification of exposure situations if women changed their residences during pregnancy. Furthermore, data were not available for other potential confounders including specific maternal characteristics (education, occupation), exposures during pregnancy (diet, medication use, smoking, alcohol), health conditions (diabetes), and genetic factors. Lastly, data on pregnancy termination before 28 weeks of gestation were not included in this study, which may lead to underestimation of the occurrence of congenital heart disease.

In conclusion, our study showed that exposure to PM_2.5_ in gestational weeks 6 to 27 was associated with an increased risk of congenital heart disease in offspring with birth defects, male offspring exhibited heightened susceptibility. Further fundamental experimental research is needed to explore the mechanisms underlying this phenomenon. We suggest that relevant departments continue to strengthen the work of air pollution control while formulating health protection and intervention strategies for pregnant and postpartum women against air pollution, reducing the occurrence of birth defects and improving the quality of the population.

## Data Availability

The raw data supporting the conclusions of this article will be made available by the authors, without undue reservation.
